# The utility of serum markers in the conservative management of placenta accreta spectrum (PAS) by leaving the placenta in situ in women with high‐grade PAS

**DOI:** 10.1002/ijgo.71113

**Published:** 2026-06-01

**Authors:** Ammar Al Naimi, Charlotte Wiesmann, Doerthe Brueggmann, Anna Elisabeth Hentrich, Eileen Deuster, Samira Catharina Hoock, Lukas Jennewein, Frank Louwen

**Affiliations:** ^1^ Department of Obstetrics and Prenatal Medicine Goethe‐University Hospital Frankfurt Germany

**Keywords:** biomarkers, PAS, placenta accreta spectrum, placenta in situ

## Abstract

**Objective:**

To investigate the association between serum markers and successful conservative management of patients with placenta accreta spectrum (PAS).

**Methods:**

This was a retrospective case–control study where patients with high‐grade PAS between 2011 and 2025 undergoing conservative leaving the placenta in situ were included. The management followed the Frankfurt PAS Procedure where the placenta was left in situ after cesarean section (CS) followed by uterine artery embolization and two‐step surgery. Regular laboratory investigations included the assessment of serum markers including beta human chorionic gonadotropin (β‐hCG), serum placental growth factor (PlGF), progesterone, and pregnancy‐associated plasma protein A (PAPP‐A). Mixed effects models with random intercept were utilized to assess the association between the marker levels and uterine preservation over follow‐up time.

**Results:**

The study included 24 women, seven of which with successful uterine preservation had lower β‐hCG, PlGF and progesterone with medians of 1469 (282.2, 5532), 117 (27.2, 258), and 24.5 (4.2, 66) compared to the 17 needing hysterectomy with medians of 9041 (3563, 16 824), 227.2 (129, 368), and 60 (47, 83), respectively, but no difference in PAPP‐A levels. The mixed effects models showed a decline for β‐hCG, PAPP‐A, progesterone over time (*P* < 0.001), but not for PlGF. The rate of β‐hCG drop was higher for patients with preserved uterus especially within the first two weeks of follow‐up with a coefficient of 311 mIU/mL per day.

**Conclusion:**

β‐hCG, PlGF and progesterone levels successively decrease after conservative PAS management, and there is a negative association between successful uterine retention and serum β‐hCG levels. The optimal window for performing a two‐step surgery might be 2–3 weeks post CS.

## 1 INTRODUCTION

Placenta accreta spectrum (PAS) represents a heterogeneous range of conditions associated with abnormally adherent placenta that leads to severe blood loss. The apposition of the placental trophoblast to a scarred myometrium, where defects in the decidua basalis are present, is key to the development of the condition, and high‐grade PAS is associated with significant maternal morbidity and mortality. The traditional classification based on the depth of the placental villi relative to the uterine wall is outdated, and current research focuses on investigating the utero‐placental interface to understand the underlying pathophysiology.^1^, ^2^ PAS constitutes an increasingly prevalent clinical problem with parallel rising numbers to cesarean sections worldwide. The prevalence for PAS ranges from one in 533 to one in 730 deliveries, and the once considered rare clinical condition with incidence around one in 4000 births in the 1970s, showed a 10‐fold rise to become a common risk for women's health.^3^, ^4^, ^5^

The topicality of PAS is evidenced by the growing number of publications on the subject^3^, ^4^ as well as the integration into national and international guidelines.^6^, ^7^ Cesarean hysterectomy is considered the gold standard for the management of high‐grade PAS cases and care should only be provided by multidisciplinary teams in centers of excellence to improve patient safety.^8^

There are other options to treat PAS and avoid hysterectomy which are considered conservative approaches in PAS surgeries. These include leaving the placenta in situ untouched after delivery, ligating the umbilical cord with absorbable suture material, closing the uterotomy and the abdominal wall, and waiting for the expulsion or complete resorption of the placenta due to necrosis which may take months to occur. This procedure could be offered to women who are able to attend follow‐up, have a quick hospital access, and adamant to try and preserve their uterus.^9^ Moreover, this approach could be lifesaving in resource‐limited settings for women with unexpected intrapartum PAS diagnosis as a first step before a two‐step surgery can be appropriately planned after optimizing preparedness.^10^ Additional measures for reducing uterine blood flow and accelerating placental detachment such as uterine artery embolization could be attempted.^11^ Intravenous antibiotics for 24 h followed by a further 4 days of oral antibiotics, and low molecular weight heparin for 10 days are recommended for women with a placenta left in situ. This approach could reduce the risk of blood loss by half and the risk of iatrogenic injuries by one third compared to cesarean hysterectomy.^8^, ^12^, ^13^

Successful management with fertility preservation has previously been reported in 78% of women with low‐ to high‐grade PAS.^9^ High‐level evidence for leaving the placenta in situ in patients with a high‐grade PAS is sparse. The largest review of 60 cases reported successful uterine preservation in 60% of women and a risk of 42% for major complications such as sepsis or coagulopathy cases.^14^ Tissue necrosis, sepsis, acute bleeding, fistula, venous thromboembolism, and acute renal failure are some of the risks that these women could face. Moreover, long follow‐up, due to unknown best time for a second surgery in order to remove the placenta, is a disadvantage of the procedure. Therefore, this approach remains experimental.^9^, ^15^, ^16^, ^17^

Furthermore, there is no evidence for adjusting the follow‐up period for women with high‐grade PAS who are managed this way, that could decrease the risk of complications such as septicemia or hemorrhage. Increased blood loss might be linked to neovascularization and reperfusion of the placenta, and there might be an optimal window for removing the placenta prior to neovascularization. The current literature provides limited evidence for predictive biomarkers that can be clinically employed to show neoangiogenesis in the placental unit.^18^ There is a potential role for serum placental growth factor (PlGF), progesterone, beta human chorionic gonadotropin (β‐hCG) and pregnancy‐associated plasma protein A (PAPP‐A) levels in identifying this window.^19^, ^20^ Therefore, the biomarkers have the potential to assist in determining the safe period for two‐step surgery after initial referral. The aim of this study was to investigate an association between the serum markers and successful conservative management of patients with high‐grade PAS.

## 2 MATERIALS AND METHODS

This retrospective case–control study, based on a prospectively followed‐up cohort, was a single center study conducted at the Department of Obstetrics and Prenatal Medicine of the hospital of Goethe University of Frankfurt between 2011 and 2025. Patients with antenatal PAS diagnosis based on sonographic and magnetic resonance imaging (MRI) findings were accordingly counseled.^21^, ^22^ Women who decided to attempt conservative management through leaving the placenta in situ were included in this study after providing informed consent. Exclusion criteria were under age women below 18 years, false‐positive high‐grade PAS identified intraoperatively, women opting for planned cesarean hysterectomy, and nonconsenting unwilling women. The International Federation of Gynecology and Obstetrics (FIGO) criteria for intraoperative PAS diagnosis were utilized to identify high‐grade PAS.^23^ The management of these patients followed the standardized protocol of the Frankfurt PAS Procedure (FPP) where the placenta was left in situ after cesarean section (CS) and uterine artery embolization followed. FPP surgery is performed via an infra‐umbilical midline skin incision, the baby is delivered via a vertical fundal uterine incision, and the placenta is left in situ after the umbilical cord is ligated close to its placental insertion site. After closure, women are directly transferred to the interventional radiology, where standardized uterine artery embolization is performed. All patients receive prophylactic antibiotic therapy for 4 days before ending in‐patient care.

Follow‐up is conducted in an outpatient setting, where ultrasound, clinical examination, and bloodwork are performed weekly. This includes the measurement of progesterone with progesterone ELISA enzyme immunoassay,^24^ PIGF with Elecsys PlGF, β‐hCG with Elecsys hCG + β electrochemiluminescence immunoassay, and PAPP‐A with Elecsys PAPP‐A electrochemiluminescence immunoassay.^25^ The decision to perform two‐step surgery to remove the placenta, initially with trial of hysteroscopic resection or followed with laparotomy and focal uterine resection, was based on clinical findings and not on the acquired serum markers. Electronic medical records were used to acquire the demographic variables. The main outcome was the difference in progesterone, PIGF, β‐hCG, and PAPP‐A levels between patients depending on successful IRP and uterine preservation. Hemoglobin levels after end of follow‐up and the second surgery, transfused blood units, and length of stay in the intensive care unit (ICU) were assessed as secondary outcomes and served as surrogates for maternal short‐term morbidity. Mixed effects model with random intercept were utilized to assess the association between the levels of the serum markers on one hand and successful uterine preservation and follow‐up time. A *P* value less than 0.05 was considered statistically significant and all statistical analyses were conducted using Stata (version 18, Texas, USA). The present study was approved by the local ethics committee.

## 3 RESULTS

The study period included 24 women with intraoperatively diagnosed high‐grade PAS who underwent IRP. The likelihood of successful FPP was 29.2% whereas 17 women required hysterectomy at time of second surgery. The mean maternal age was 35.3 ± 4.1 years, gestational age at diagnosis was 28.7 ± 5.9 weeks, and FPP was performed at 34.4 ± 2.8 gestational week. Placenta previa was present in 83.3% of patients, but more likely to be associated with requiring hysterectomy 94.1% than with successful FPP 57.1%. There were no differences in the number of previous cesarean sections, myomectomy or curettage between women with successful and unsuccessful FPP. Successful FPP was associated with lower blood loss, 0.2 L (interquartile range [IQR]: 0.2–2) of blood, compared to unsuccessful FPP, 4 L (IQR: 1.8–6) of blood. Nevertheless, there were no differences in the postoperative hemoglobin levels post‐hysterectomy, 7.4 g/dL (IQR: 6.8–8.1) compared with post placental removal 7.5 g/dL (IQR: 6.8–8.2). The mean length of stay at the ICU was extremely limited with 0.13 ± 0.35 days and there was no statistically significant difference between women with successful and unsuccessful FPP. Patients with successful uterine preservation had lower (*P* < 0.05) β‐hCG, PlGF and progesterone with medians of 1469 (282.2, 5532), 117 (27.2, 258), and 24.5 (4.2, 66) compared to patients needing hysterectomy with medians of 9041 (3563, 16 824), 227.2 (129, 368), and 60 (47, 83), respectively. There was no difference between PAPP‐A levels for patients with successful uterine preservation 65.2 (28.1, 100.59) compared with patients needing hysterectomy 54.1 (46.65, 102.065) with *P* value of 0.49. A summary of the differences between the demographic characteristics and the investigated outcomes for women with successful FPP compared to requiring hysterectomy is presented in Table 1.

**TABLE 1:** The demographic characteristics and outcomes of the study participants.

	Preserved uterus (*N* = 7)	Required hysterectomy (*N* = 17)	*P* value
Age (years)	33.29 (4.50)	35.35 (4.31)	0.30
Gestational age at diagnosis (weeks)	28.71 (6.50)	27.06 (6.35)	0.57
Placenta previa	4 (57.14%)	16 (94.12%)	0.027
Number of previous CS			
0	3 (42.86%)	2 (11.76%)	0.26
1	3 (42.86%)	8 (47.06%)	
2	1 (14.29%)	3 (17.65%)	
3	0 (0.00%)	4 (23.53%)	
Number of previous D&C			
0	2 (28.57%)	9 (52.94%)	0.43
1	4 (57.14%)	4 (23.53%)	
2	1 (14.29%)	3 (17.65%)	
3	0 (0.00%)	1 (5.88%)	
History of myomectomy	1 (14.29%)	1 (5.88%)	0.50
Gestational age at delivery	33.83 (2.99)	34.67 (2.87)	0.60
Stay in intensive care (days)	0.00 (0.00)	0.22 (0.44)	0.24
Delta PlGF (%)	12 (3–23)	40 (25–56)	0.018
Delta progesterone (%)	6 (1–25)	20 (13–25)	0.13
Delta hCG (%)	1 (0–2)	4 (2–9)	0.018
Delta PAPP‐A (%)	20 (7–37)	1 (0–41)	0.49
Total blood loss (mL)	200 (200–2000)	4000 (1800–6000)	0.051
Hemoglobin level day 1 post‐hysterectomy or placenta‐removal (g/dL)	7.5 (6.8–8.2)	7.4 (6.8–8.1)	0.91

Abbreviations: CS, cesarean section; D&C, dilation and curettage; hCG, human chorionic gonadotropin; PAPP‐A, pregnancy‐associated plasma protein A; PlGF, serum placental growth factor.

The mixed effects models (Tables 2, 3, 4, 5) showed a statistically significant decline for β‐hCG, PAPP‐A, progesterone over follow‐up period (*P* < 0.001), but the mean change in PlGF over time was not statistically significant (*P* 0.26). Moreover, the rate of β‐hCG drop was higher for patients with preserved uterus than patients requiring hysterectomy especially within the first two weeks of follow‐up with a coefficient of 311 mIU/mL per day (*P* < 0.001). The trends of serum marker change for the two groups are shown in Figures 1, 2, 3, 4.

**TABLE 2:** Mixed‐effects regression for the association between β‐hCG and successful FPP and time.

Variable	Coefficient (95% CI)	*P* value
Successful FPP (reference hysterectomy)	−10 316 (−15 399, −5233)	<0.001
Postoperative follow‐up (days)	−593 (−691, −495)	<0.001
Interaction‐term for successful FPP # postoperative follow‐up (reference hysterectomy)	311 (157, 464)	<0.001
Intercept	17 436 (13 997, 20 875)	<0.001

Abbreviations: β‐hCG, beta human chorionic gonadotropin; CI, confidence interval; FPP, Frankfurt PAS Procedure.

**TABLE 3:** Mixed‐effects regression for the association between PlGF and successful FPP and time.

Variable	Coefficient (95% CI)	*P* value
Successful FPP (reference hysterectomy)	262 (−265, 789)	0.33
Postoperative follow‐up (days)	−5.25 (−14.37, 3.86)	0.26
Interaction‐term for successful FPP # postoperative follow‐up (reference hysterectomy)	−6.44 (−21.02, 8.13)	0.39
Intercept	329.46 (−26.13, 685.05)	0.069

Abbreviations: CI, confidence interval; FPP, Frankfurt PAS Procedure; PlGF, serum placental growth factor.

**TABLE 4:** Mixed‐effects regression for the association between progesterone and successful FPP and time.

Variable	Coefficient (95% CI)	*P* value
Successful FPP (reference hysterectomy)	−38.63 (−74.50, −2.75)	0.035
Postoperative follow‐up (days)	−2.25 (−3.08, −1.41)	<0.001
Interaction‐term for successful FPP # postoperative follow‐up (reference hysterectomy)	0.32 (−1.06, 1.69)	0.654
Intercept	97.57 (73.56, 121.58)	<0.001

Abbreviations: CI, confidence interval; FPP, Frankfurt PAS Procedure.

**TABLE 5:** Mixed‐effects regression for the association between PAPP‐A and successful FPP and time.

Variable	Coefficient (95% CI)	*P* value
Successful FPP (reference hysterectomy)	27.92 (0.66, 55.19)	0.045
Postoperative follow‐up (days)	−2.09 (−2.74, −1.43)	<0.001
Interaction‐term for successful FPP # postoperative follow‐up (reference hysterectomy)	−0.57 (−1.35, 0.21)	0.153
Intercept	84.62 (60.54, 108.69)	<0.001

Abbreviations: CI, confidence interval; FPP, Frankfurt PAS Procedure; PAPP‐A, pregnancy‐associated plasma protein A.

**FIGURE 1:**
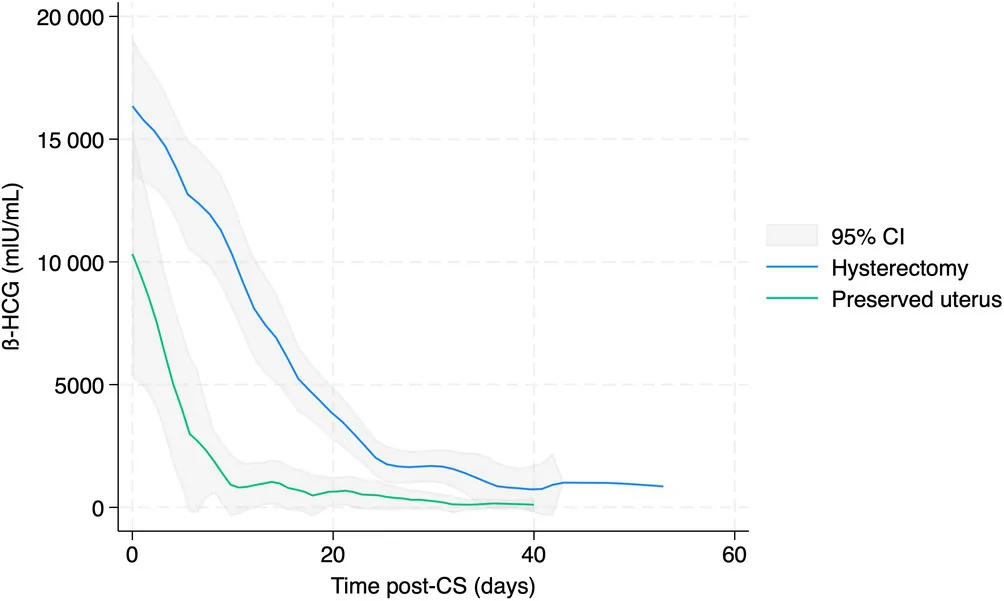
Two‐way plot of the estimated beta‐human chorionic gonadotropin (β‐hCG) over time stratified by Frankfurt PAS Procedure (FPP) success and uterine preservation. CI, confidence interval.

**FIGURE 2:**
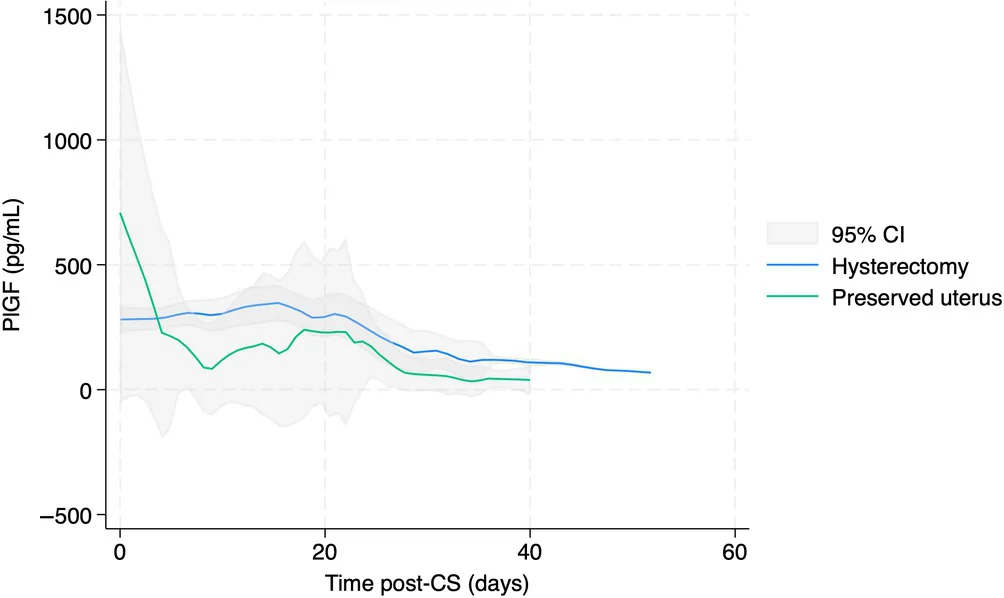
Two‐way plot of the estimated serum placental growth factor (PlGF) over time stratified by Frankfurt PAS Procedure (FPP) success and uterine preservation. CI, confidence interval.

**FIGURE 3:**
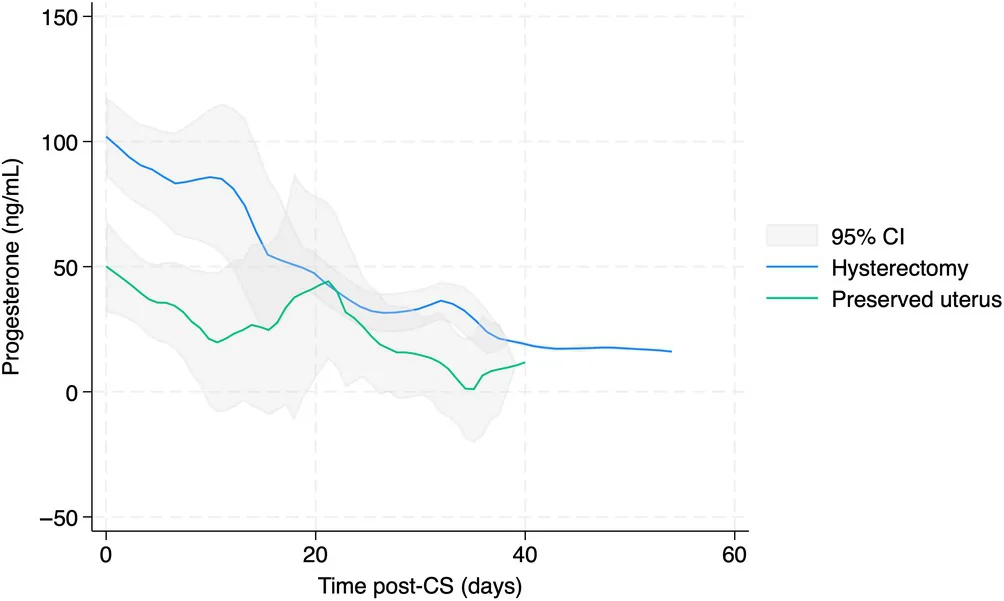
Two‐way plot of the estimated progesterone over time stratified by Frankfurt PAS Procedure (FPP) success and uterine preservation. CI, confidence interval.

**FIGURE 4:**
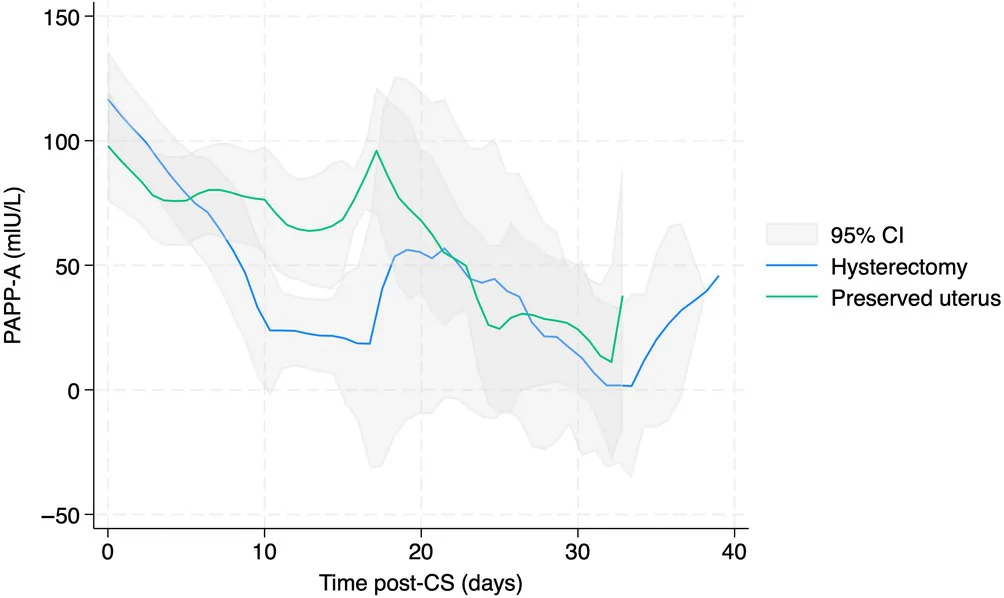
Two‐way plot of the estimated pregnancy‐associated plasma protein A (PAPP‐A) over time stratified by Frankfurt PAS Procedure (FPP) success and uterine preservation. CI, confidence interval.

## 4 DISCUSSION

The present study showed that β‐hCG, PlGF and progesterone levels successively decrease after conservative PAS management. Moreover, a negative association between successful uterine retention and serum β‐hCG levels was shown. Therefore, this work could be considered a pilot study to understand changes in serological markers after leaving the placenta in situ, and could be utilized for better planning of the two‐step intervention for these patients.

The present study investigated four potential biomarkers that are associated with placentation and angiogenesis. PIGF is a potent angiogenic glycoprotein produced by the placenta and extravillous trophoblast. An upregulation of angiogenic growth factors like PIGF, VIGF or angiopeptide‐2 along with a simultaneously reduced expression of antiangiogenic proteins as PIGF‐receptor or soluble fms‐like tyrosine kinase‐1 has been observed in patients with PAS.^26^ Studies showed that PlGF levels after leaving the placenta in situ decrease, and this change seems to correlate with localized angiogenesis in PAS.^27^ Interestingly PlGF levels showed a sharp drop within the first week for those with preserved uterus, but then there was no significant change over time. Therefore, even if PlGF could not be utilized to assess the optimal time for second surgery, a drastic primary fall after IRP could be predictive of successful uterine preservation. PlGF might be a sign of reperfusion.

Progesterone on the other hand starts in the ovarian granulosa cells and is then taken over by the placental syncytiotrophoblast cells after the eighth gestational week to reach a peak at term with about 300 mg of progesterone per day.^28^ Progesterone levels kept decreasing over our follow‐up period with a steeper slope within the first 3 weeks. Furthermore, women with a preserved uterus had lower progesterone levels than those requiring hysterectomy.

β‐hCG is a glycopeptide hormone that is detectable in maternal serum 10 days after ovulation. Next to its major role in maintaining the function of corpus luteum by the alpha subunit acting as a luteinizing hormone super agonist, the beta subunit is involved in auto‐ and paracrine mechanisms regulating the cytotrophoblast differentiation and the implantation and development of the placenta.^29^ Several publications have shown pregnancy‐related changes in β‐hCG serum levels in women with PAS.^19^, ^20^ Our study showed that β‐hCG levels drop after FPP and women with preserved uterus have significantly lower levels as well as significantly faster drop. The β‐hCG decrease in patients with successful FPP occurred within the first two weeks before it plateaued. This might indicate beginning of placental revascularization which leads to maintained β‐hCG levels or β‐hCG decrease could be an indicator of successful embolization.

PAPP‐A is a zinc metalloprotease that is produced by placental syncytiotrophoblasts in increasing concentration during pregnancy. It regulates the local bioavailability of insulin‐like growth factor (IGF) by cleaving the inhibitory insulin growth factor binding proteins (IGFBP‐4/‐5).^30^ This controls the uptake and transport of amino acids and glucose into the trophoblastic cells and promotes the auto‐ and paracrine mediated trophoblastic invasion.^31^ Increased levels of PAPP‐A in the first trimester have already demonstrated an association with PAS.^19^, ^20^ Our data showed gradual decrease in PAPP‐A over time after FPP, but the extent of PAPP‐A fall did not differ between women with preserved uterus and women requiring hysterectomy. This could indicate that PAPP‐A has no role in neoangiogenesis and revascularization of the placenta and therefore might not be useful in predicting successful FPP.

β‐hCG, PlGF and progesterone levels successively decrease after conservative PAS management. Moreover, there is a statistically significant negative association between successful conservative PAS management with retained uterus and serum β‐hCG levels. The biggest β‐hCG drop occurs within two weeks of CS, and there is a short‐time increase in PlGF and progesterone level between the second and third postoperative week. Therefore, 2–3 weeks post CS could be the optimal window for performing a secondary surgery.

This study had several strengths such as the use of FIGO criteria for diagnosing high‐grade PAS. To the best of our knowledge, there is no published data investigating the study question. Therefore the present study provides important information regarding potential biomarkers in the management of PAS. Nevertheless, our results have to be interpreted in light of some limitations. This pilot study was limited by a small number of patients and thus our results are preliminary. Any lack of statistical significance in our study could be attributed to the limited sample size instead of real biological indifference. Lastly, the examined sample size limited our capability to conduct adjusted analyses to explore potential confounders such as the presence of placenta previa.

## 5 CONCLUSION

This study shows that FPP is a safe procedure for managing patients with high‐grade PAS. Despite the fact that blood loss significantly increases when hysterectomy is required, this did not affect the hemoglobin levels of patients after hysterectomy compared to placental removal. This is probably caused by adequate compensation and management with blood transfusion.

We recommend a multicenter trial to determine the relevance of serum β‐hCG and PIGF levels after leaving the placenta in situ for individual timing of two‐step intervention.

## AUTHOR CONTRIBUTIONS

FL, AA and CW conceived and designed the study. LJ and CW managed the data. AA, CW, and DB performed data analysis and wrote the first version of the manuscript. All authors contributed revisions, edits, and approved the manuscript for submission. All authors had full access to all the data in the study and accept responsibility for the decision to submit for publication. AEH, ED and SCH verified the underlying data of the study.

## CONFLICT OF INTEREST STATEMENT

All other authors declare no competing interests.

## Data Availability

The dataset generated and analyzed during the current study is available from the corresponding author upon request.
